# Multi-Objective Optimization of Damage Volume and CO_2_ Consumption for High-Pressure Liquid CO_2_ Jet Impact on Hydroxyl-Terminated Polybutadiene Propellant

**DOI:** 10.3390/ma19112354

**Published:** 2026-06-02

**Authors:** Zhen Zhang, Dayong Jiang, Yun Bai, Huidong Zhang, Yuhui Ding

**Affiliations:** 1 College of Equipment Management and Support, Engineering University of PAP, Xi’an 710086, China; 13287661653@163.com; 2 Military Basic Education College, Engineering University of PAP, Xi’an 710086, China; wanghe717@163.com; 3 State Key Laboratory Cultivation Base for Gas Geology and Gas Control, Henan Polytechnic University, Jiaozuo 454000, China; zhanghuidong@hpu.edu.cn; 4 College of Chemistry, Nankai University, Tianjin 300071, China; dingyuhui2024@163.com

**Keywords:** liquid CO_2_ jet, hydroxyl-terminated polybutadiene propellant, response surface methodology, non-dominated sorting genetic algorithm II, multi-objective optimization

## Abstract

High-pressure liquid CO_2_ jets possess the characteristics of low-temperature cooling and dry, residue-free impact, which makes this technology particularly suitable for removing hydroxyl-terminated polybutadiene (HTPB) propellant from decommissioned solid rocket motors. However, existing studies lack multi-objective optimization of impact efficiency and CO_2_ consumption, which limits their engineering applications and further promotion. In this study, a high-accuracy quadratic Response Surface Methodology (RSM) relating process parameters to damaged volume was established using a Box–Behnken design (BBD) combined with three-dimensional topography scanning. A theoretical model for CO_2_ consumption was developed based on the Homogeneous Equilibrium Model (HEM). On this basis, the Non-dominated Sorting Genetic Algorithm II (NSGA-II) was used to obtain the Pareto-optimal set for maximizing propellant damaged volume and minimizing CO_2_ consumption. The results indicate that nozzle diameter has the most significant effect on damaged volume and exhibits a strong interaction with jet pressure. The knee-point solution gives a jet pressure of 15.35 MPa, a stand-off distance of 5 mm, and a nozzle diameter of 1.8 mm. Compared with the initial condition, this compromise condition increases the damaged volume by 72% while increasing CO_2_ consumption by only 4.9%. Furthermore, the temperature in the impact zone was reduced to a minimum of −92.4 °C, with no thermal accumulation observed. These findings reveal the influence of liquid CO_2_ jet process parameters on impact efficiency and CO_2_ consumption, providing a theoretical basis and parameter references for its engineering application in the safe removal of propellants from decommissioned solid rocket motors.

## 1. Introduction

The safe and efficient removal of hydroxyl-terminated polybutadiene (HTPB) propellant from decommissioned solid rocket motors is a prerequisite for high-value casing recovery and propellant resource utilization [1,2,3]. Currently, although high-pressure water jet technology is the mainstream removal method, the large volume of wastewater containing perchlorate ions generated during treatment exacerbates the burden on environmental management [4]. Furthermore, water jet impact readily generates explosive dust aerosols, posing a risk of accidental ignition or detonation in enclosed spaces [5]. By contrast, liquid carbon dioxide (CO_2_) jets exhibit strong potential for energetic material removal because of their chemical inertness, low-temperature cooling, and dry, residue-free erosion characteristics [6,7].

Previous studies have shown that jet pressure, stand-off distance, and nozzle diameter are the key process parameters affecting the erosion of HTPB propellant by liquid CO_2_ jets [8]. However, current studies evaluate erosion performance only by penetration depth and damaged area, which fails to accurately reflect the actual amount of propellant removed. Moreover, existing work focuses only on single-objective improvement of impact efficiency and neglects CO_2_ consumption. In practical engineering applications, pursuing the maximum damaged volume alone requires a substantial increase in jet pressure or nozzle diameter, which inevitably causes a sharp rise in CO_2_ consumption. Conversely, excessive restriction on CO_2_ consumption sacrifices removal efficiency. These two objectives are inherently competitive. Therefore, systematic multi-objective optimization is urgently needed to clarify the relationship between erosion efficiency and CO_2_ consumption.

Response Surface Methodology (RSM) can construct a regression surrogate model between process parameters and response with a limited number of experiments. It can also capture interactions among factors. Therefore, it has been widely used for parameter optimization in various manufacturing processes [9,10,11]. Among these methods, the Box–Behnken design (BBD) requires fewer experiments and excludes extreme vertex combinations in the design space, thereby ensuring the accuracy of quadratic polynomial modeling while effectively avoiding the potential safety risks of extreme operating conditions, which makes it particularly suitable for experiments involving energetic materials [12,13]. However, this study must simultaneously address the mutually constraining objectives of maximizing the damaged volume and minimizing CO_2_ consumption. For such a multi-objective optimization problem, the desirability function built into RSM depends on subjective weighting and thus lacks objectivity. Moreover, each weight set can only yield one compromise solution, failing to reveal the full trade-off relationship between the objectives [14]. The Non-dominated Sorting Genetic Algorithm II (NSGA-II) maintains population diversity and converges to a uniformly distributed Pareto front within a finite number of iterations through fast non-dominance sorting. When combined with RSM, it avoids the subjectivity of RSM while reducing the computational cost of NSGA-II. This integrated method has shown significant advantages in multi-objective optimization and has been effectively applied to parameter optimization in various fields [15,16,17,18,19]. It is therefore well suited to the multi-objective optimization of HTPB propellant erosion by liquid CO_2_ jets.

For theoretical modeling of CO_2_ consumption, liquid CO_2_ undergoes a rapid pressure drop and flash evaporation inside the nozzle, causing its flow behavior to deviate significantly from the ideal gas assumption. The Homogeneous Equilibrium Model (HEM) assumes that the gas and liquid phases remain in local thermodynamic equilibrium and has been shown to predict the critical mass flow rate of liquid CO_2_ under throttling conditions with high accuracy [20,21,22]. Combined with the NIST REFPROP thermophysical property database, it enables reliable quantitative calculation of CO_2_ consumption under different operating conditions and thus provides a theoretical basis for constructing the cost objective. For damaged volume characterization, the flash evaporation behavior of liquid CO_2_ jets result in highly irregular erosion pit topographies, which leads to large errors in conventional two-dimensional geometric approximation methods. Three-dimensional topography scanning technology, based on high-precision point cloud acquisition and surface reconstruction, can directly and accurately determine the actual material removal volume and effectively address the limitations of traditional evaluation metrics [23].

To clarify the trade-off between the erosion performance of liquid CO_2_ jets on HTPB propellant and CO_2_ consumption, this study analyzed the influence of process parameters on the erosion volume using BBD experimental design combined with 3D topography scanning technology, and established a response surface model for predicting the erosion volume. A calculation model for CO_2_ consumption was developed by the HEM. The NSGA-II algorithm was then used to obtain the Pareto-optimal set for the two objectives. Combined with validation under representative operating conditions and temperature monitoring, the reliability and safety of the optimization results were comprehensively evaluated. The findings are expected to provide systematic parameter selection criteria and engineering guidance for the safe and efficient removal of HTPB propellant from decommissioned solid rocket motors.

## 2. Materials and Methods

### 2.1. Experimental Materials and Setup

This experiment used a typical four-component HTPB propellant consisting of ammonium perchlorate, aluminum, HTPB binder, and hexogen. All specimens had a uniform thickness of 53 mm. At room temperature, the propellant exhibited a tensile strength of 4 MPa, a compressive strength of 10 MPa, a shear strength of 108 MPa [24], and a glass transition temperature of about −70 °C.

**Figure 1:**
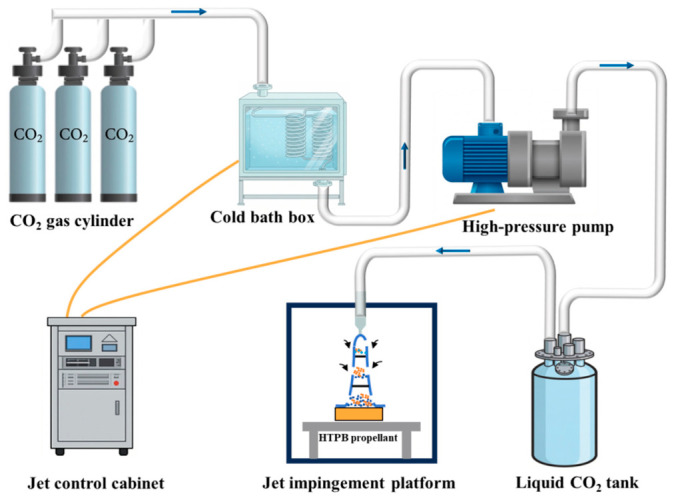
High-pressure liquid CO_2_ jet impingement test system.

The experiment was conducted using a self-developed high-pressure liquid CO_2_ impact test platform. The overall system layout and the experimental setup are shown in Figure 1. The system consists of a CO_2_ gas cylinder, a cold bath box, a high-pressure pump, a liquid CO_2_ tank, a control cabinet, and an impingement platform. During the experiment, gaseous CO_2_ was delivered to the cold bath box. It was cooled and converted into liquid CO_2_. The liquid CO_2_ was then pressurized by the high-pressure pump and stored in the liquid CO_2_ tank as high-pressure liquid CO_2_. Finally, the liquid CO_2_ in the tank was ejected at high speed through the nozzle and impacted the HTPB propellant specimen placed inside the explosion-proof chamber. The jet control cabinet precisely controlled the impact pressure and impact time. Each test was repeated three times, and the average value was used as the final result. After the experiment, the residual CO_2_ in the vessel was transferred through a recovery pipeline to a purification and recirculation system. After passing through two-stage debris separators and a purifier, it is re-liquefied and stored in the CO_2_ storage tank. This process reduced operating cost and ensured experimental safety and environmental protection.

### 2.2. Experimental Design

#### 2.2.1. Selection of Experimental Variables

In the experiment, pressure (P), stand-off distance (S), and nozzle diameter (D) were selected as the primary factors. Preliminary experiments showed that when the jet pressure was below 10 MPa, the jet failed to cause significant damage to the propellant. When the pressure exceeded 40 MPa, the jet penetrated the propellant. This penetration caused the jet’s kinetic energy to be transferred to the back of the target, preventing the formation of an erosion pit suitable for quantitative measurement, thereby affecting the assessment of the actual erosion volume and subsequent modeling optimization.

Therefore, a pressure range of 15–35 MPa was selected as the effective erosion range. For nozzle diameter, values below 1 mm provide insufficient jet impact capacity, whereas larger diameters hinder the rapid pressurization of liquid CO_2_ by the equipment. Therefore, the nozzle diameter was set at 1–2 mm. Because liquid CO_2_ undergoes violent expansion and phase change in the atmosphere and is sensitive to stand-off distance, experimental observations indicate that when the stand-off distance exceeds 10 mm, the core zone disperses significantly and the effective impact dynamic pressure drops sharply. Therefore, the stand-off distance was set at 5–10 mm. The impact duration was fixed at 8 s, considering both the impact effect and the pressure-supply characteristics of the equipment, with experiments conducted under standard atmospheric pressure. Due to the irregularity of the damaged regions, using either impact depth or damage area alone has limitations in quantifying the damage effect. Therefore, this study selects the removed volume as the metric for erosion effect and CO_2_ consumption as the cost indicator.

#### 2.2.2. BBD Experimental Design

The BBD is a commonly used experimental design method in RSM. Compared to full factorial designs, BBD can establish a quadratic polynomial model with high predictive accuracy using fewer experiments. Unlike the Central Composite Design (CCD), BBD excludes combinations at the design space vertices, ensuring good identification of main effects and interaction effects while offering better economic efficiency [25].

In this study, HTPB propellant is subject to potential combustion or detonation under high-impact loads. Therefore, the experimental design must balance effective coverage of the parameter space with the predictive capability of the regression model, while minimizing safety risks from extreme factor combinations. The BBD excludes extreme vertex combinations in the design space, effectively avoiding potential hazards under extreme operating conditions and offering strong engineering applicability and experimental safety. BBD is a typical three-factor design, with each factor set at low, medium, and high levels. The design includes 12 edge-centered experiments and 5 central-point experiments, totaling 17 runs. The factors and their coded levels are listed in Table 1.

#### 2.2.3. Statistical Analysis

In this study, Design-Expert 13.0 software was used for BBD modeling and statistical analysis. Analysis of Variance (ANOVA) was employed to test the overall significance of the regression model and examine the individual coefficients, with a significance level of α = 0.05. The F-test and *p*-value were used to evaluate the main effects, quadratic effects, and interactions of each factor. The model’s goodness of fit and predictive ability were evaluated using the lack-of-fit test and the coefficient of determination R^2^. For model validation, the residual normal probability plot, scatter plot of residuals versus predicted values, and comparison plot of predicted versus observed values were used to assess the model’s systematic bias and prediction accuracy.

### 2.3. Experimental Methods

**Figure 2:**
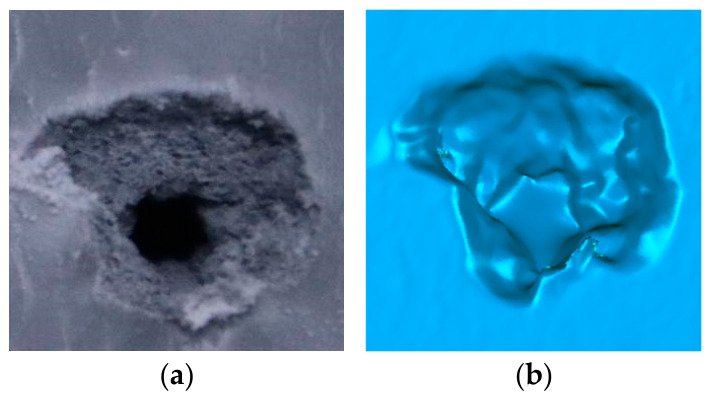
Damaged morphology: (**a**) Morphology of erosion pit Erosion pit; (**b**) 3D reconstruction morphology.

To quantify the fragmentation effect of liquid CO_2_ jets impacting on solid propellant, a 3D surface scanner was used to measure the removed volume. It is an OptimScan 5 M high-precision blue-light 3D inspection system manufactured by SHINING 3D (Hangzhou, China). The measurement range was 100 mm × 75 mm, and the measurement accuracy was 0.005 mm. The flat surface of the unaffected peripheral region was taken as the reference plane. After the impact test, a panoramic scan of the same end face of the specimen was conducted to capture the 3D point cloud data, including the erosion pits. The point cloud was then imported into Geomagic Wrap software 2021 for denoising, meshing, and surface reconstruction. Through coordinate alignment, the enclosed volume between the reconstructed surface and the reference plane was calculated as the absolute removal volume. To reduce random errors, each operating condition was repeated three times, and the average value was used as the final removal volume. The repeated center-point tests in the BBD were further used to evaluate the pure experimental error. The relative standard deviation was approximately 7.23%, which indicates that the measurements had acceptable repeatability. In this experiment, the propellant damage morphology and its corresponding 3D topography scan are shown in Figure 2.

## 3. Results

### 3.1. Results of BBD Experiments

In accordance with the BBD scheme described in Section 2.2.2, a total of 17 experiments were conducted within the three levels of jet pressure, stand-off distance, and nozzle diameter. After each experiment, a 3D topography scanner was used to reconstruct the erosion pits on the propellant samples and calculate their removed volumes. The experimental conditions and corresponding response results are shown in Table 2.

A quadratic polynomial regression model was fitted to the erosion volume using Design-Expert 13.0. To facilitate comparison of factor effects and eliminate dimensional differences, the regression analysis was performed using coded variables, where A, B, and C represent jet pressure, stand-off distance, and nozzle diameter, respectively. The resulting quadratic polynomial is as follows.

(1) $$\begin{array}{l} V=159.4500+81.7163A-27.5863B+235.4850C-28.9325AB\\ +50.4700AC-20.1100BC+1.8963A^{2}-7.4238B^{2}+118.4188C^{2} \end{array}$$

### 3.2. RSM Statistical Analysis of Erosion Volume

#### 3.2.1. ANOVA of Model

To quantitatively evaluate the effects of jet pressure, stand-off distance, and nozzle diameter on the erosion volume of HTPB propellant, ANOVA was used to assess the overall significance of the regression model and each regression coefficient, with the significance level set at α = 0.05. The results are shown in Table 3.

The results show that the model F-value was 380.63 with *p* < 0.0001, indicating that the established quadratic regression model was highly significant and could adequately describe the response relationship between the process parameters and the erosion volume of HTPB propellant within the investigated factor ranges. The *p*-value for the model’s lack of fit term was 0.3179, which is greater than 0.05, indicating good reliability of the model fit. In addition, R^2^ = 0.9979, demonstrating an excellent fit of the regression model.

For the main effects, all three factors were significant, with the influence ranked as C>A>B. For the interaction effects, AB, AC, and BC were all significant, and AC showed the strongest interaction, indicating that the combination of nozzle diameter and jet pressure had the most sensitive effect on erosion volume. For the quadratic terms, only $C^{2}$ was significant, indicating a strong nonlinear relationship between nozzle diameter and erosion volume, whereas $A^{2}$ and $B^{2}$ were not significant, suggesting that the effects of jet pressure and stand-off distance on erosion volume were dominated by linear terms within the investigated parameter range. It should be noted that the RSM modeling was not established for a single factor. It was developed to describe the variation in propellant removal volume under the combined effects of the three factors. The subsequent interaction analysis and multi-objective optimization were also carried out based on this three-factor model.

#### 3.2.2. Analysis of Residuals

**Figure 3:**
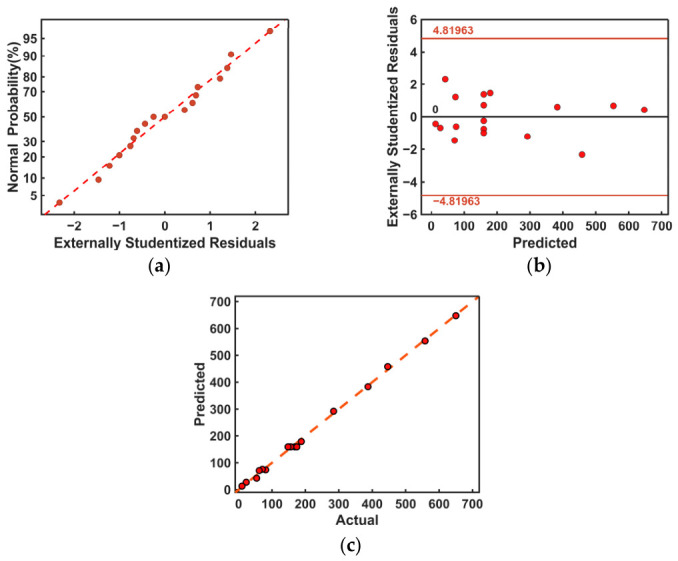
Residual diagnostics for the removal volume RSM: (**a**) normal probability distribution of residuals (**b**) distribution of residuals and predicted values (**c**) distribution of predicted and actual values.

The results of ANOVA indicate that the established model is statistically reliable. Residual diagnostics further verify its goodness of fit and predictive capability. As shown in Figure 3a, the experimental points in the normal probability plot of residuals are distributed close to the reference line, with no obvious deviation or abnormal outliers. This indicates that the model residuals approximately follow a normal distribution and that the model shows no significant systematic bias in describing the erosion volume of HTPB propellant under liquid CO_2_ jet impact. In Figure 3b, the residuals are randomly distributed with respect to the predicted values and all fall within the limits of ±4.81963, with no clear trend of dispersion or abnormal fluctuation. This indicates that the prediction error is stable and that the model can reliably reflect the effects of process parameters on erosion volume. In Figure 3c, the predicted values agree well with the measured values, and most data points are located near the 45° reference line. This demonstrates that the established quadratic response surface model has a good fitting performance and can provide a reliable basis for subsequent process parameter optimization. In summary, the results in Figure 3 show that the model performs well in terms of residual normality, error randomness, and agreement between predicted and measured values. This indicates that the model can reliably describe the combined effects of jet pressure, stand-off distance, and nozzle diameter on propellant removal volume. It further confirms that the established model has good statistical validity and engineering applicability.

#### 3.2.3. Interaction Analysis

**Figure 4:**
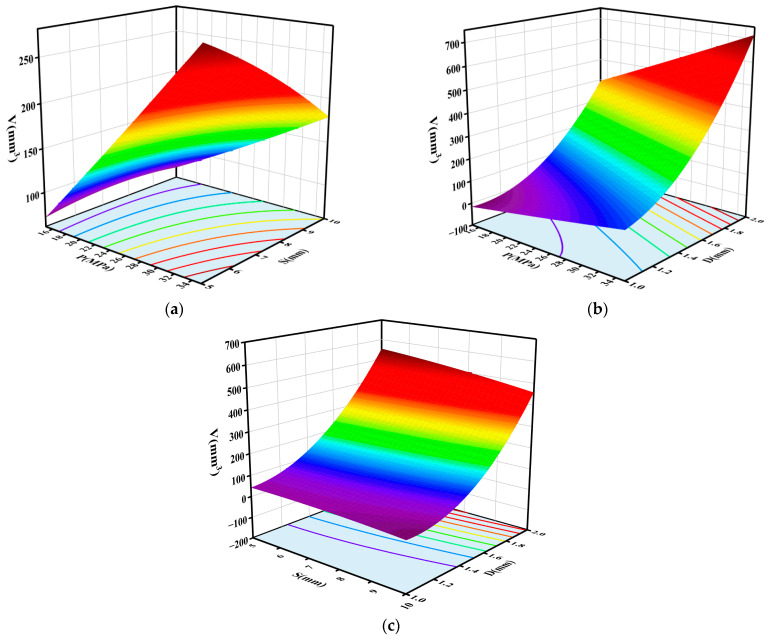
Response surfaces and contour plots for interactions on removal volume: (**a**) the interaction surface between P and S; (**b**) the interaction surface between P and D; (**c**) the interaction surface between S and D.

Based on the above regression Equation (1), one factor was fixed at its center level, and the three-dimensional response surfaces and contour plots of the pairwise interactions on erosion volume were generated, as shown in Figure 4. As shown in Figure 4a, there is a significant interaction between A and B on V. Within the high-pressure range, the erosion volume decreased with increasing stand-off distance, indicating that a larger stand-off distance weakened the erosion effect of the high-pressure jet. This is mainly because the high-pressure liquid CO_2_ jet undergoes rapid expansion, phase change, and entrainment diffusion after leaving the nozzle. As the stand-off distance increases, the velocity in the jet core region decays, and the effective impact load on the target surface decreases [26,27]. Therefore, a smaller stand-off distance is more favorable for maintaining impact concentration and energy utilization efficiency.

Figure 4b shows a highly significant interaction between A and C. As jet pressure and nozzle diameter increased simultaneously, the erosion volume of material increased markedly. Under a larger nozzle diameter, the promoting effect of jet pressure on erosion volume became more pronounced. This is because a larger nozzle diameter not only increases the CO_2_ mass flow rate per unit time, but also expands the jet’s effective area. Higher pressure increases the jet velocity and enables the jet to generate higher shear stress near the target surface [28,29]. Under their combined effect, crack initiation, propagation, and local spallation of the propellant are more likely to occur, thereby significantly enhancing the volume removal capacity. In contrast, under low-pressure conditions, the increase in flow rate caused by a larger nozzle diameter alone is difficult to fully convert into effective damage, and thus its promoting effect is therefore relatively limited.

In addition, Figure 4c shows that although a certain interaction exists between B and C, it is not particularly pronounced overall, and its effect is clearly weaker than that of AB and AC. In general, the erosion volume increased with nozzle diameter, whereas its response to stand-off distance was relatively moderate. This indicates that, under this parameter combination, the variation in erosion volume was still mainly governed by the main effect of nozzle diameter, while stand-off distance acted more as a secondary factor affecting jet propagation and the erosion process rather than a decisive factor. Therefore, to increase the erosion volume, priority should be given to the coordinated matching of jet pressure and nozzle diameter, while the stand-off distance should be maintained within a reasonable range.

### 3.3. Construction of the Theoretical Model for CO_2_ Consumption

To quantitatively characterize CO_2_ consumption during jet discharge, the total CO_2_ consumption was defined as the time integral of the nozzle mass flow rate.

(2) $$M_{C}=\int_{0}^{t}\dot{m}(t)dt$$

where $M_{C}$ is the total CO_2_ consumption, kg, and $\dot{m}(t)$ is the nozzle mass flow rate at time *t*, kg/s. In this study, high-pressure liquid CO_2_ undergoes throttling and rapid depressurization in the nozzle, which induces phase transition. When the downstream back pressure is lower than the critical pressure, the nozzle throat reaches a choked state. Under this condition, the mass flow rate at the throat no longer increases as the downstream pressure decreases. It reaches the critical mass flow rate for the given operating condition. Therefore, the mass flow rate can be approximated by the critical mass flow rate, and Equation (2) can be simplified as

(3) $$M_{C}={\dot{m}}_{c}t$$

${\dot{m}}_{c}$ is the critical mass flow rate corresponding to the inlet conditions. Due to rapid pressure drop and phase change of high-pressure liquid CO_2_ inside the nozzle, the ideal gas equation of state cannot accurately describe its thermodynamic properties in the phase-change region. To ensure the accuracy of thermophysical properties, the NIST REFPROP database was used, and the Span–Wagner equation of state was applied to calculate thermodynamic parameters such as enthalpy, entropy, and density of CO_2_. Considering the entire flow process of liquid CO_2_, the flow from the storage tank to the nozzle outlet can be divided into upstream pipeline transport and rapid expansion within the nozzle. In this experimental system, the inner diameter of the upstream pipeline is 10 mm and its total length is 4 m, while the nozzle outlet diameter is only 1–2 mm. Based on the Darcy–Weisbach equation [30,31], with a pipe wall relative roughness of 0.002, the Reynolds number under operating conditions of 15–35 MPa is approximately 10^5^, corresponding to a friction factor of about 0.018. Under the 15–35 MPa operating conditions in this study, the frictional pressure drop in the upstream pipeline does not exceed 1% of the tank pressure, which is far smaller than the local pressure drop caused by the nozzle throttling. Therefore, the effect of upstream pressure loss can be neglected, and the nozzle inlet condition can be approximated as the initial state of CO_2_ in the storage tank.

The flow inside the nozzle is described by the HEM, assuming local thermodynamic equilibrium between gas and liquid phases with identical velocity, pressure, and temperature. Based on one-dimensional steady, adiabatic, and isentropic assumptions, the mass flow rate at the nozzle throat is expressed as

(4) $$\dot{m}=\frac{\pi D^{2}}{4}\rho_{x}u_{x}$$

where D is the nozzle diameter, m, $\rho_{x}$ are the densities of the homogeneous fluid in the throat, kg/m^3^, $u_{x}$ is the flow velocity in the throat, m/s.

Under adiabatic and isentropic conditions, total enthalpy and entropy are conserved from the nozzle inlet to the throat [32].

(5) $$h_{0}+\frac{u_{0}^{2}}{2}=h_{x}+\frac{u_{x}^{2}}{2}$$

(6) $$s_{0}=s_{x}$$

where $h_{0}$ and $s_{0}$ are the enthalpy J/kg and entropy J/(kg·K) at the nozzle inlet, respectively, $h_{x}$ and $s_{x}$ are the enthalpy and entropy of the throat state, respectively, $u_{0}$ is the flow velocity at the nozzle inlet. Since the inlet velocity is much smaller than the throat velocity, the inlet kinetic energy can be neglected, and the throat velocity can be expressed as (7).

(7) $$u_{x}=\sqrt{2(h_{0}-h_{x})}$$

For a given throat pressure, the vapor quality $x_{t}$ under gas–liquid equilibrium can be determined from $s_{0}=s_{x}$, and the corresponding $h_{x}$ and $\rho_{x}$ can be obtained, as shown in (8) and (9).

(8) $$h_{x}=x_{t}h_{g}+(1-x_{t})h_{l}$$

(9) $$\rho_{x}={\left[\frac{x_{t}}{\rho_{g}}+\frac{1-x_{t}}{\rho_{l}}\right]}^{-1}$$

where $h_{g}$ and $h_{l}$ are the specific enthalpies of saturated vapor and liquid, $\rho_{g}$ and $\rho_{l}$ are their densities. Thus, Equation (4) can be expressed as.

(10) $$\dot{m}=\frac{\pi D^{2}}{4}\rho_{x}\sqrt{2(h_{0}-h_{x})}$$

According to two-phase critical flow theory, when choked flow occurs, the mass flow rate at the throat reaches a maximum and becomes independent of downstream back pressure. The critical mass flow rate is given by (11).

(11) $${\dot{m}}_{c}=\underset{p_{r}\leq p_{t}\leq p_{0}}{\max}\dot{m}$$

where $p_{0}$ is the nozzle inlet pressure, $p_{r}$ is the triple-point pressure of CO_2_. Since the model is based on gas–liquid equilibrium, pressures below the triple point may lead to gas–solid or gas–liquid–solid states, which are beyond the model scope. Therefore, the throat pressure is limited to the range between $p_{r}$ and $p_{0}$.

**Figure 5:**
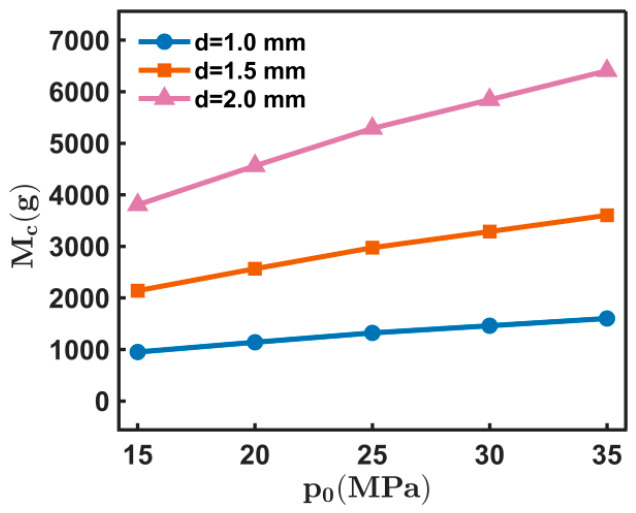
CO_2_ consumption with different conditions.

In the numerical solution process, this paper uses MATLAB R2024b to access the REFPROP databa $T_{0}$ se to calculate the inlet conditions $h_{0}$ and $s_{0}$ of the nozzle for given $p_{0}$ and. The throat pressure was then discretely scanned between $p_{0}$ and $p_{r}$ [33,34]. At each pressure node, the corresponding $\rho_{x}$ and $h_{x}$ are solved based on $s_{0}=s_{x}$, and the mass flow rate at that pressure is calculated using Equation (10). The maximum value among all nodes was taken as the critical mass flow rate for that operating condition. Substituting this value into Equation (3) yields the total CO_2_ consumption during the quasi-steady jetting stage under the given condition. The relationship between jet parameters and total CO_2_ consumption is shown in Figure 5.

## 4. Multi-Objective Optimization Based on NSGA-II Algorithm

### 4.1. Pareto Front Solution and Its Distribution Characteristics

**Figure 6:**
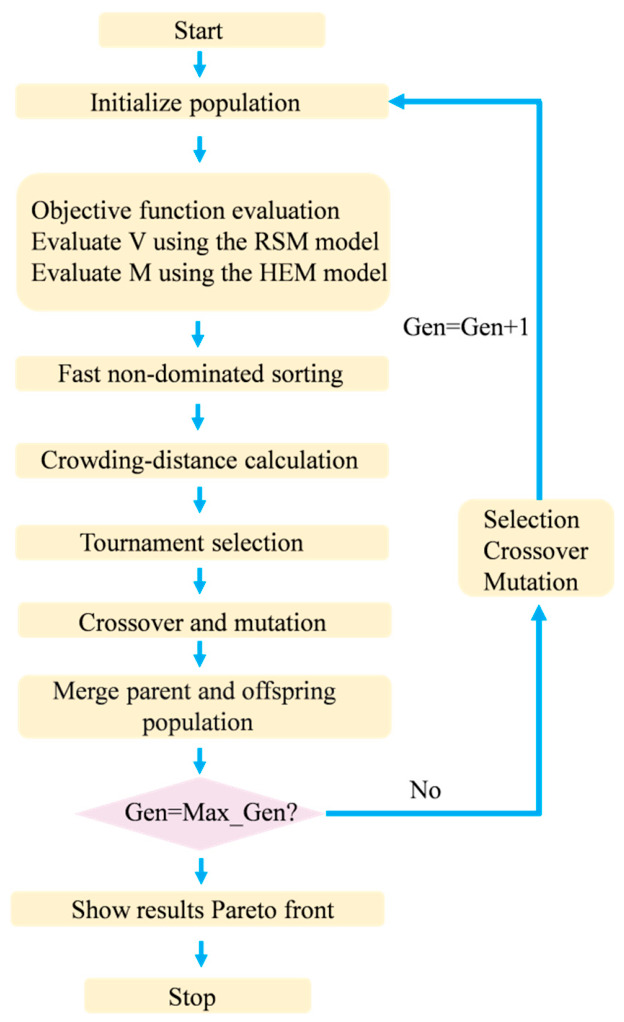
The multi-objective optimization procedure of NSGA-II.

Based on the RSM of removed volume established in Section 3 and the HEM for CO_2_ consumption, the NSGA-II algorithm was employed to perform two-objective optimization of the process parameters for liquid CO_2_ jet erosion of HTPB propellant. The algorithm is based on Pareto dominance and applies fast non-dominated sorting to stratify the population. It incorporates a crowding-distance metric to maintain solution diversity. This approach shows strong applicability and robustness in handling complex engineering problems with competing objectives [35,36]. In the encoding process, the three input variables P, S, and D form the gene segments of each chromosome, while the removed volume and CO_2_ consumption are defined as conflicting objectives. Unlike single-parameter optimization, this method yields a set of non-dominated feasible solutions. It provides a more comprehensive basis for condition selection under different engineering preferences. The solution procedure is shown in Figure 6.

**Figure 7:**
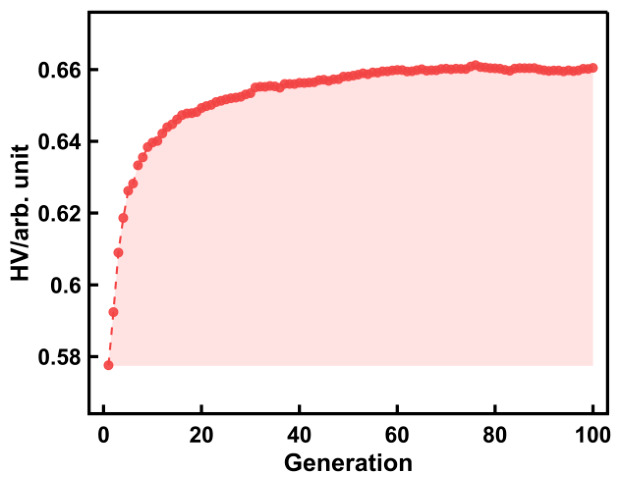
HV convergence curve of the Pareto solution set.

**Figure 8:**
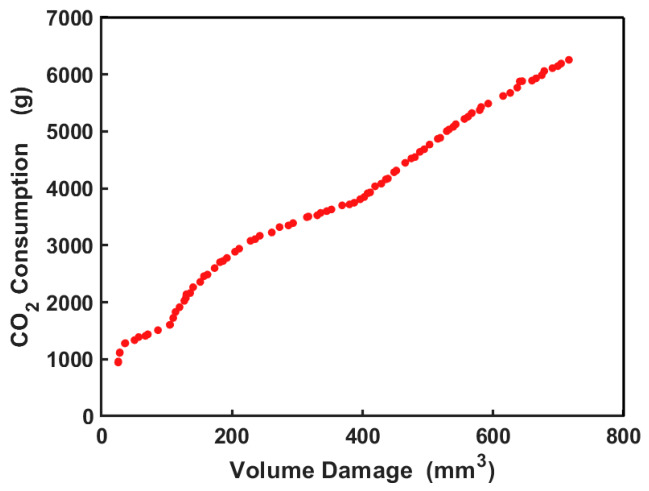
Pareto frontier.

During the solution process, the ranges of the three decision variables were restricted to 15–35 MPa, 5–10 mm, and 1–2 mm, respectively. The condition V>0 was imposed to eliminate nonphysical negative predictions of the surrogate model near the design-space boundaries. The initial population size of NSGA-II was set to 100, with crossover and mutation probabilities set to 0.9 and 0.1, respectively, and the maximum number of iterations set to 100. The Hypervolume (HV) metric was introduced to evaluate the overall quality of the non-dominated solution set in each generation [37], and the results are shown in Figure 7. The HV increases rapidly in the early iterations and then gradually slows. It stabilizes after about 60 generations, and further iterations beyond 100 generations yield only minimal improvements. This indicates that a stable Pareto solution set can be obtained under the current parameter settings. The final Pareto front is shown in Figure 8. The front exhibits a monotonically increasing arc, indicating a clear trade-off between removed volume and CO_2_ consumption; increasing the removed volume inevitably raises CO_2_ consumption, and vice versa.

### 4.2. Selection and Validation of Representative Parameter Sets

To systematically investigate the erosion behavior of HTPB propellant under different impact intensities of liquid CO_2_ jets and to characterize the overall features of the multi-objective optimization results, three representative non-dominated solutions were selected from the Pareto-optimal set obtained by NSGA-II for validation experiments. The experimental conditions were consistent with the BBD experiments, with removed volume as the primary evaluation metric. Each condition was repeated three times, and the measured results were averaged. Since CO_2_ consumption was calculated using the HEM, no independent measurements were conducted in this section, and it was used only for economic comparison under different conditions. The center-point condition $K_{0}$ in the BBD design space was introduced as the baseline for the initial design. Its inclusion in the validation experiments aims to evaluate the improvement achieved by the multi-objective optimization relative to the initial design.

The knee-point method was used to select the Group B parameters from the Pareto front. This method first performs dimensionless transformation on the objective functions, calculates the orthogonal distance from each non-dominant solution in the computational space to the line connecting the two ends of the Pareto front, and finally selects the solution with the largest orthogonal distance as the knee point. This point balances marginal gain and marginal cost between the two objectives and represents the best compromise solution [38]. Based on this, a high removal-volume condition (Group A) and a low CO_2_-consumption condition (Group C) were selected from the two ends of the Pareto front. The validation results of the typical conditions are shown in Table 4.

From Table 4, the relative errors between the measured and RSM-predicted removal volumes for Groups A, B, and C are 2.54%, 11.18%, and 10.46%, respectively, all within 15%. Group A shows the smallest error, indicating high predictive accuracy in the high removal-volume region. Although Groups B and C exhibit slightly larger errors, the measured trends are consistent with the optimization direction. Considering system errors of the experimental equipment and material heterogeneity, these deviations are acceptable. The above validation results indicate that the quadratic RSM established in Section 3 can accurately describe the quantitative relationship between process parameters and propellant removal volume. The NSGA-II multi-objective optimization results based on this surrogate model are therefore reliable.

Based on the above experimental results, a systematic comparison was conducted between Case B and the initial scheme K_0_. In Case K_0_, the jet pressure, stand-off distance, and nozzle diameter were 25.00 MPa, 7.50 mm, and 1.50 mm, respectively, with a measured removal volume of 155.82 mm^3^ and CO_2_ consumption of 2919.20 g. Compared with K_0_, Case B increases the removal volume by approximately 72% while CO_2_ consumption rises by only 4.9%, and the jet pressure decreases by 38.6%. These results indicate that the optimized scheme achieves a substantial improvement in removal volume with minimal additional resource consumption and enhanced operational safety, demonstrating the effectiveness of the proposed multi-objective optimization method.

### 4.3. Safety Verification of Typical Operating Conditions

**Figure 9:**
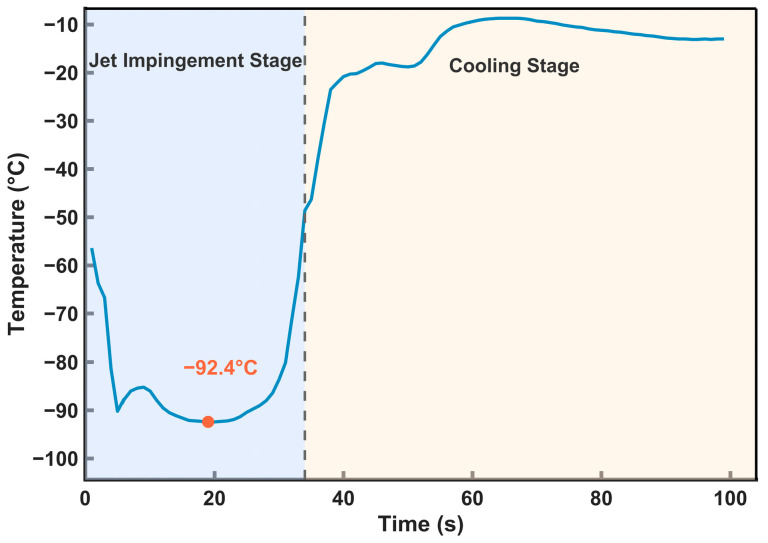
Surface temperature of the propellant under case B during 30 s impact.

During liquid CO_2_ jet impact on HTPB propellant, mechanical energy is converted into thermal energy. Local hotspots and temperature rise may occur in the impact region, posing safety risks. Studies indicate that the process temperature for HTPB propellant removal should not exceed 280 °C [39,40]. Therefore, the temperature evolution on the propellant surface during impact is a key indicator for evaluating the safety of liquid CO_2_ jet processing. To verify the thermal safety of the liquid CO_2_ jet erosion process, condition B was selected with an impact duration of 30 s. A T thermocouple was used to monitor the surface temperature in real time. The temperature–time curve is shown in Figure 9.

As shown in Figure 9, when the jet struck the propellant surface, the temperature rapidly decreases to about −56 °C, then continues to drop and stabilizes near −90 °C, with a minimum value of −92.4 °C. After the impact, the surface temperature rises rapidly at first and then recovers gradually. No abnormal secondary temperature peak is observed, and no thermal accumulation occurs during the entire process. This is because the high-pressure liquid CO_2_ in the storage tank undergoes a sudden drop in pressure after throttling and expansion through the nozzle, resulting in a significant Joule-Thomson effect and a marked decrease in temperature. When the nozzle outlet pressure falls below the CO_2_ triple-point pressure, flash evaporation occurs, and part of the liquid rapidly converts into a mixture of solid dry ice particles and gaseous CO_2_. During flash evaporation, latent heat absorption further decreases the temperature, allowing the jet temperature at the propellant surface to drop below −78.5 °C [41,42]. In addition, the jet medium after nozzle expansion contains a large number of solid dry ice particles. When these particles impact the propellant surface, they undergo sublimation and further absorb heat from the impact zone, producing a sustained cryogenic cooling effect [43]. This cooling continuously imposes forced convective cooling on the impact zone, promptly removing the frictional heat generated by mechanical impact and suppressing temperature rise. As a result, the cooling effect of the jet far exceeds the frictional heating effect, and the propellant surface temperature remains below −56 °C throughout the process, which is far below the safe temperature for HTPB propellant removal.

## 5. Conclusions

This study addressed the trade-off between impact efficiency and CO_2_ consumption in the high-pressure liquid CO_2_ jet impact process for HTPB propellants. We established a RSM for predicting the volume of damage and a HEM calculation model for calculating CO_2_ consumption. Furthermore, we utilized the NSGA-II algorithm to achieve multi-objective of impact efficiency and CO_2_ consumption. The main conclusions are as follows:

(1) The Pareto front obtained by multi-objective optimization revealed the trade-off between the damaged volume and CO_2_ consumption. The comprehensive compromise operating parameters selected by the knee-point method was a jet pressure of 15.35 MPa, a stand-off distance of 5 mm, and a nozzle diameter of 1.8 mm. Validation experiments showed that, compared with the initial condition, this compromise condition increased the damaged volume by 72% while increasing CO_2_ consumption by only 4.9%, and reduced the jet pressure by about 38.6%.
(2) A quadratic response surface model with high fitting accuracy was established based on the BBD experiments, and RSM was used to analyze the effects of various factors on the response values. The results of the ANOVA indicate that nozzle diameter has the most significant effect on the damaged volume, and that there is a strong interaction between nozzle diameter and jet pressure. In process parameter optimization, jet pressure and nozzle diameter should be coordinated first, while the stand-off distance should be properly controlled to maintain jet energy density.
(3) Thermal safety tests showed that, during liquid CO_2_ jet impingement, the propellant surface temperature dropped to a minimum of −92.4 °C, and no heat accumulation occurred throughout the process. This confirms the excellent safety of this technology for HTPB propellant removal.

Moreover, this study was limited to a single HTPB propellant formulation, and the applicability of the conclusions to other solid propellants requires further validation. The idealized assumptions introduced in the HEM may cause some discrepancy between the theoretical and actual CO_2_ consumption. Future work may incorporate two-phase flow numerical simulations to further improve the predictive accuracy of the model.

## Figures and Tables

**Table 1:** BBD factors and coded levels.

Factors	Factors Level		
P	−1	0	1
S	−1	0	1
D	−1	0	1

**Table 2:** BBD experimental conditions and removal-volume response values.

Number	P (MPa)	S (mm)	D (mm)	V (mm^3^)
1	15	5	1.5	62.05
2	35	5	1.5	284.5
3	15	10	1.5	81.21
4	35	10	1.5	187.93
5	15	7.5	1	10.02
6	35	7.5	1	71.36
7	15	7.5	2	387.23
8	35	7.5	2	650.45
9	25	5	1	54.26
10	25	10	1	22.84
11	25	5	2	558.27
12	25	10	2	446.41
13	25	7.5	1.5	174.53
14	25	7.5	1.5	147.81
15	25	7.5	1.5	150.34
16	25	7.5	1.5	168.21
17	25	7.5	1.5	156.36

**Table 3:** ANOVA of the quadratic RSM for removal volume.

Source	Sum of Squares	df	Mean Square	F-Value	*p*-Value	
Model	5.776 × 10^5^	9	64,179.36	380.63	<0.0001	significant
A	53,420.36	1	53,420.36	316.82	<0.0001	
B	6088.01	1	6088.01	36.11	0.0005	
C	4.436 × 10^5^	1	4.436 × 10^5^	2631.03	<0.0001	
AB	3348.36	1	3348.36	19.86	0.0029	
AC	10,188.88	1	10,188.88	60.43	0.0001	
BC	1617.65	1	1617.65	9.59	0.0174	
A^2^	15.14	1	15.14	0.0898	0.7731	
B^2^	232.05	1	232.05	1.38	0.2791	
C^2^	59,044.21	1	59,044.21	350.18	<0.0001	
Residual	1180.29	7	168.61			
Lack of Fit	648.12	3	216.04	1.62	0.3179	not significant
Pure Error	532.17	4	133.04			
Cor Total	5.788 × 10^5^	16				

**Table 4:** Validation results of representative operating conditions.

Case	P (MPa)	S (mm)	D (mm)	Theoretical Damage Volume (mm^3^)	Actual Damage Volume (mm^3^)	Relative Error (%)	CO_2_ Consumption (g)
A	35.00	5.00	2.00	716.64	698.45	2.54	6259.78
B	15.35	5.00	1.80	241.37	268.36	11.18	3061.24
C	15.00	10.00	1.00	27.06	24.23	10.46	943.20

## Data Availability

The original contributions presented in this study are included in the article. Further inquiries can be directed to the corresponding author.
